# Meconium in the Amniotic Fluid of Pregnancies Complicated by Preterm Premature Rupture of Membranes Is Associated With Early Onset Neonatal Sepsis

**DOI:** 10.1155/S1064744995000251

**Published:** 1995

**Authors:** Michael J. Kupferminc, Elizabeth Wickstrom, Nam H. Cho, Patricia M. Garcia

**Affiliations:** 1 Section of Maternal-Fetal Medicine Department of Obstetrics and Gynecology Northwestern University Medical School Northwestern Memorial Hospital Chicago IL USA; 2 Prentice Women's Hospital 333 E. Superior Street Suite 410 Chicago IL 60611 USA

## Abstract

*Objective:* This study was to determine the significance of meconium in the amniotic fluid of pregnancies complicated by preterm premature rupture of membranes (PPROM) without labor.

*Methods:* A case-control study of 31 pregnancies complicated by PPROM at 27–36 weeks gestation with meconium present (study group) and 93 pregnancies complicated by PPROM but without meconium was performed. The patients were matched for year of delivery, gestational age, race, and parity. Pregnancy and neonatal outcome variables of the 2 groups were compared.

*Results:* The incidence of early onset neonatal sepsis was significantly increased in the study group (16.1% vs. 1.1%; *P* < 0.001). Similarly, chorioamnionitis (48.3% vs. 22.5%; *P* < 0.01), cesarean delivery for a nonreassuring fetal heart rate pattern (19.4% vs. 3.2%; *P* < 0.01), a 5-min Apgar score < 7 (22.5% vs. 8.6%; *P* < 0.05), and fetal growth retardation (FGR) (12.9% vs. 2.2%; *P* < 0.05) were also more common in pregnancies complicated by PPROM with meconium. The mean umbilical cord arterial pH was significantly lower in these pregnancies (7.18 ± 0.07 vs. 7.28 ± 0.08; *P* < 0.001). After controlling for confounding variables with multiple logistic regression analysis, we found that meconium in the amniotic fluid remained associated with early onset neonatal sepsis.

*Conclusions:* The presence of meconium in the amniotic fluid of pregnancies complicated by PPROM is associated with an increased incidence of early onset neonatal group B β-hemolytic streptococcus (GBBS) sepsis.


Infectious Diseases in Obstetrics and Gynecology 3:22–27 (1995)
© 1995 Wiley-Liss, Inc.

### Meconium in the Amniotic Fluid of Pregnancies Complicated by Preterm Premature Rupture of Membranes Is Associated With Early Onset Neonatal Sepsis

**Michael J. Kupferminc, Elizabeth Wickstrom, Nam H. Cho, and Patricia M. Garcia**

*Section of Maternal-Fetal Medicine, Department of Obstetrics and Gynecology, Northwestern University Medical School, Northwestern Memorial Hospital, Chicago, IL*

### Abstract

***Objective:*** **This study was to determine the significance of meconium in the amniotic fluid of pregnancies complicated by preterm premature rupture of membranes (PPROM) without labor.**

***Methods:*** **A case-control study of 31 pregnancies complicated by PPROM at 27–36 weeks gestation with meconium present (study group) and 93 pregnancies complicated by PPROM but without meconium was performed. The patients were matched for year of delivery, gestational age, race, and parity. Pregnancy and neonatal outcome variables of the 2 groups were compared.**

***Results:*** **The incidence of early onset neonatal sepsis was significantly increased in the study group (16.1% vs. 1.1%; $P < 0.001$). Similarly, chorioamnionitis (48.3% vs. 22.5%; $P < 0.01$), cesarean delivery for a nonreassuring fetal heart rate pattern (19.4% vs. 3.2%; $P < 0.01$), a 5-min Apgar score < 7 (22.5% vs. 8.6%; $P < 0.05$), and fetal growth retardation (FGR) (12.9% vs. 2.2%; $P < 0.05$) were also more common in pregnancies complicated by PPROM with meconium. The mean umbilical cord arterial pH was significantly lower in these pregnancies (7.18 ± 0.07 vs. 7.28 ± 0.08; $P < 0.001$). After controlling for confounding variables with multiple logistic regression analysis, we found that meconium in the amniotic fluid remained associated with early onset neonatal sepsis.**

***Conclusions:*** **The presence of meconium in the amniotic fluid of pregnancies complicated by PPROM is associated with an increased incidence of early onset neonatal group B β-hemolytic streptococcus (GBBS) sepsis.** © 1995 Wiley-Liss, Inc.

Key words

Neonatal infection, intraamniotic infection, group B β-hemolytic streptococcus

Preterm premature rupture of membranes (PPROM) is directly related to 30–40% of the cases of preterm delivery.[1] The etiology of PPROM is most likely multifactorial, but appears to be strongly related to infection,[2] specifically with group B β-hemolytic streptococcus (GBBS).[3–5] Infrequently, PPROM is associated with the appearance of meconium, as the presence of meconium in the amniotic fluid is considered a part of the maturation process of the gastrointestinal tract.[6] Consequently, the significance of meconium in preterm pregnancies with or without PPROM is unknown. Several reports suggest an association between meconium and microbial invasion of the amniotic cavity in preterm pregnancies and between the presence of meconium and neonatal infection.[7–10] To address the significance of this relationship, we investigated the outcomes of pregnancies complicated by

**Address correspondence to Dr. Patricia M. Garcia, Prentice Women's Hospital, 333 E. Superior Street, Suite 410, Chicago, IL 60611.**

Received June 28, 1994

Accepted March 7, 1995

Clinical Study

PPROM with and without meconium in the amniotic fluid.

### SUBJECTS AND METHODS

The perinatal data base of Northwestern Memorial Hospital from 1986 to 1992 was reviewed to identify patients with PPROM associated with meconium-stained amniotic fluid but without regular uterine contractions at the time of admission. The presence or absence of meconium-stained amniotic fluid was recorded from the physician's admitting note. Forty-two patients were identified, and the maternal and neonatal charts were reviewed by the authors (M.J.K., E.W.). The gestational age was determined by reliable menstrual dates or first- or second-trimester ultrasound examination and was confirmed by Ballard examination of the neonate. Eleven pregnancies were excluded from the study because of discrepancies in gestational age assignment which suggested the pregnancy might be >36 weeks gestation. Thirty-one pregnancies with PPROM associated with meconium-stained amniotic fluid remained and were analyzed; 3 of these pregnancies were twin gestations and only twin A was included in the study. The control group consisted of 93 nonlaboring singleton pregnancies complicated by PPROM without meconium. Using a 3:1 matching scheme, we selected the charts in the perinatal data base of consecutive pregnancies for the PPROM associated with meconium-stained amniotic fluid group and matched these patients for year of delivery, gestational age at delivery, parity, and race.

During the study period, all pregnancies complicated by PPROM with or without meconium were managed expectantly, awaiting spontaneous labor. Cervicovaginal cultures for GBBS were obtained upon admission. Signs of maternal infection or fetal compromise prompted an induction of labor. Treatment prior to labor for a positive GBBS cervicovaginal culture was left to the discretion of the attending physician. The patients with positive cultures received ampicillin or erythromycin intrapartum. Beginning in 1989, the patients with unknown culture status also received intrapartum prophylactic antibiotics.

Demographic and outcome variables were abstracted from the maternal and neonatal medical records. The latency period was calculated as the time from documented rupture of membranes to the time of delivery. Oligohydramnios was noted if the largest vertical pocket of amniotic fluid was <2 cm or if the total amniotic fluid index was <5 cm. Chorioamnionitis was defined as a maternal temperature ≥38°C with uterine tenderness and sustained fetal tachycardia. The route of delivery and indication for cesarean delivery were recorded from the patient's chart. Endometritis was defined as a fever of ≥38°C with uterine tenderness in any 2 of the first 10 days postpartum, exclusive of the first 24 h.

The diagnosis of early onset neonatal sepsis was made if there were clinical symptoms of sepsis in association with positive blood or cerebrospinal-fluid cultures within 72 h of birth. Fetal growth retardation (FGR) was defined as a birth weight less than the tenth percentile for gestational age, as determined by Brenner et al.[11] Respiratory distress syndrome (RDS) was diagnosed based upon the observation of chest retractions, grunting, cyanosis, a need for supplemental oxygen therapy, and a reticular pattern or air bronchograms on chest radiograph. Neonatal intraventricular hemorrhage (IVH) was defined as hemorrhage in the subependymal germinal matrix rupturing into the lateral ventricle and was diagnosed by ultrasound or computerized tomography. Only grade III and IV IVH were recorded.[12] Necrotizing enterocolitis was diagnosed based on clinical signs of abdominal distention, bloody stool, and radiographic signs of intestinal distention or pneumatosis intestinalis.

The data were analyzed using SPSS/PC+, version 5.0 (Chicago, IL). The continuous variables were analyzed by Student's t-test. Dichotomous variables were analyzed by McNemar chi-squared test and Fisher exact test. A conditional multiple logistic regression analysis was used to evaluate the effects of several potential confounding variables. The statistical significance was assumed at $P < 0.05$. A 3:1 matching scheme was used to increase the power of the study to detect a clinically significant difference between cases and controls.

### RESULTS

There was no difference between the study and control groups in the mean maternal age (27.2 ± 5.8 vs. 29.5 ± 6.1 years), mean gestational age at admission (33.2 ± 2.7 vs. 32.8 ± 2.4 weeks), parity (32.3% vs. 30.1% nulliparous), and race. However, the number of nonprivate patients

TABLE 1. Antepartum, intrapartum, and postpartum course[a]

| | Cases (N = 31) | Controls (N = 93) | P |
|---|---|---|---|
| Latency (h) | 42.6 ± 60 | 115.9 ± 139 | <0.001 |
| Oligohydramnios | 15 (48.3%) | 44 (47.3%) | NS |
| Maternal GBBS | 12/26 (46.1%) | 15/72 (20.8%) | <0.05 |
| Vaginal exams | 3.5 ± 1.5 | 3.9 ± 1.3 | NS |
| Duration of internal monitoring (h) | 3.64 ± 3.7 | 4.66 ± 4.45 | NS |
| Chorioamnionitis | 15 (48.3%) | 21 (22.5%) | <0.01 |
| Antibiotics during labor | 20 (64.5%) | 53 (56.9%) | NS |
| Chorioamnionitis treated with antibiotics during labor | 13 (86.7%) | 20 (95.2%) | NS |
| Cesarean delivery | 9 (29.0%) | 13 (14.0%) | NS |
| Cesarean delivery for nonreassuring fetal heart rate pattern | 6 (19.4%) | 3 (3.2%) | <0.01 |
| Postpartum endometritis | 8 (25.8%) | 17 (18.2%) | NS |

[a]Mean ± SD. NS, not significant.

TABLE 2. Neonatal outcome[a]

| | Cases (N = 31) | Controls (N = 93) | P |
|---|---|---|---|
| Birth weight (g) | 2,222 ± 522 | 2,256 ± 563 | NS |
| 5-min Apgar <7 | 7 (22.5%) | 8 (8.6%) | <0.05 |
| ART pH | 7.18 ± 0.07 | 7.28 ± 0.08 | <0.001 |
| SCN (days) | 11.6 ± 14.7 | 5.6 ± 8.9 | <0.05 |
| Neonatal sepsis | 5 (16.1%) | 1 (1.1%) | <0.001 |
| FGR | 4 (12.9%) | 2 (2.2%) | <0.05 |
| RDS | 7 (22.5%) | 20 (21.5%) | NS |
| IVH grade III–IV | 1 (3.2%) | 3 (3.2%) | NS |
| NEC | 1 (3.2%) | 1 (1.1%) | NS |
| Neonatal death | 2 (6.4%) | 2 (2.2%) | NS |

[a]Mean ± SD. ART pH, umbilical cord arterial pH; SCN, special-care nursery; NEC, necrotizing enterocolitis.

was significantly higher in the study group (71.0% vs. 48.4%; $P < 0.05$). The results of the pregnancies and labor courses are shown in Table 1. The latency period was significantly shorter in the PPROM associated with meconium-stained amniotic fluid group. The results of the cervicovaginal cultures for GBBS were retrievable for 26 patients (83.9%) in the PPROM associated with meconium-stained amniotic fluid group and 72 patients (77.4%) in the PPROM group. Maternal GBBS colonization, chorioamnionitis, and cesarean delivery for a nonreassuring fetal heart rate pattern were significantly more common in the PPROM associated with meconium-stained amniotic fluid group.

Table 2 summarizes the neonatal outcome variables. Early onset neonatal sepsis was significantly more common in the PPROM associated with meconium-stained amniotic fluid group. GBBS was the organism responsible for all 5 cases of neonatal sepsis in the study group and for the 1 case of neonatal sepsis in the control group. No other pathogenic organisms including *Listeria monocytogenes* were incubated from the neonates or maternal cervix. In 3 of the cases of neonatal sepsis in the PPROM associated with meconium-stained amniotic fluid group, at least 1 intrapartum dosage of antibiotics was administered >2 h before delivery for clinical signs of chorioamnionitis. In the other 2 cases of neonatal sepsis, the interval from the administration of antibiotics to delivery was <1 h because of the rapid course of labor. No intrapartum antibiotics were administered in the case of neonatal sepsis in the PPROM group. The mean umbilical cord arterial pH was significantly lower in the PPROM associated with meconium-stained amniotic fluid group, and a 5-min Apgar score <7

TABLE 3. Neonatal outcomes by gestational age groups[a]

| | <28 weeks | | | <32 weeks | | | 32–36 weeks | | |
|---|---|---|---|---|---|---|---|---|---|
| | Cases (N = 3) | Controls (N = 9) | *P* | Cases (N = 8) | Controls (N = 24) | *P* | Cases (N = 23) | Controls (N = 69) | *P* |
| Birth weight (g) | 1,206 ± 305 | 1,220 ± 423 | NS | 1,685 ± 480 | 1,687 ± 665 | NS | 2,358 ± 497 | 2,471 ± 374 | NS |
| 5-min Apgar <7 | 1 (33.3%) | 3 (33.3%) | NS | 5 (62.5%) | 4 (16.7%) | <0.05 | 2 (8.7%) | 4 (5.8%) | NS |
| ART pH | 7.16 ± 0.06 | 7.22 ± 0.05 | NS | 7.16 ± 0.08 | 7.26 ± 0.07 | <0.01 | 7.19 ± 0.06 | 7.3 ± 0.07 | <0.001 |
| SCN (days) | 47.4 ± 22.1 | 22.7 ± 10.6 | <0.05 | 25.8 ± 21.4 | 13.2 ± 9.9 | <0.05 | 7.4 ± 8.0 | 2.9 ± 5.6 | <0.05 |
| Neonatal sepsis | 1 (33.3%) | 0 | NS | 3 (37.5%) | 1 (4.2%) | <0.05 | 2 (8.7%) | 0 | 0.06 |
| FGR | 1 (33.3%) | 0 | NS | 3 (37.5%) | 1 (4.2%) | <0.05 | 1 (4.3%) | 1 (1.5%) | NS |
| RDS | 3 (100%) | 8 (88.9%) | NS | 5 (62.5%) | 17 (70.8%) | NS | 2 (8.7%) | 3 (4.34%) | NS |
| IVH grade III–IV | 1 (33.3%) | 2 (22.2%) | NS | 1 (12.5%) | 2 (8.3%) | NS | 0 | 1 (1.5%) | NS |
| NEC | 0 | 1 (11.1%) | NS | 1 (12.5%) | 1 (4.2%) | NS | 0 | 0 | NS |
| Neonatal death | 1 (33.3%) | 1 (11.1%) | NS | 2 (25%) | 2 (8.3%) | NS | 0 | 0 | NS |

[a]Means ± SD. ART pH, umbilical cord arterial pH; SCN, special-care nursery; NEC, necrotizing enterocolitis.

was more common. The incidence of FGR and the mean length of stay in the special-care nursery were also significantly greater in the PPROM associated with meconium-stained amniotic fluid group, although the neonatal mortality was similar in the 2 groups.

The apparent association between PPROM associated with meconium-stained amniotic fluid and early onset neonatal sepsis was further investigated by multiple logistic regression analysis. After we controlled for maternal GBBS colonization, latency period, chorioamnionitis, use of prophylactic antibiotics, socioeconomic status (private vs. service patient), and FGR, meconium in the amniotic fluid remained associated with neonatal GBBS sepsis (odds ratio = 11.3, 95% confidence interval [CI]: 1.2–105.0). Chorioamnionitis was the only other variable that remained associated with neonatal sepsis (odds ratio = 29.1, 95% CI: 1.6–73).

The 6 GBBS-positive patients whose neonates had early onset neonatal sepsis had a higher incidence of chorioamnionitis compared with the other 21 GBBS-positive patients (83.3% vs. 33.3%; $P < 0.05$); however, the number of nonprivate patients (100% vs. 80.1%), administration of antibiotics during labor (83.3% vs. 71.4%), and number of FGR neonates (33.3% vs. 4.8%) were comparable in these subgroups.

Table 3 summarizes the neonatal outcome variables by gestational age groups. Early onset neonatal sepsis was significantly more common in patients with PPROM associated with meconium-stained amniotic fluid who were <32 weeks gestation and approached significance in those between 32 and 36 weeks gestation. The mean umbilical cord arterial pH was significantly lower in the PPROM associated with meconium-stained amniotic fluid group <32 weeks gestation and between 32 and 36 weeks gestation.

### DISCUSSION

Our results demonstrate that PPROM associated with meconium-stained amniotic fluid is associated with early onset neonatal sepsis secondary to GBBS. Early onset neonatal sepsis in the PPROM associated with meconium-stained amniotic fluid group (16.1%) was increased appreciably over that in the control group (1.1%) and was higher than in prior studies reporting neonatal infectious morbidity following PPROM and prematurity.[13–22] Additionally, the latency period from PPROM to delivery was shorter, labor was more likely to be complicated by chorioamnionitis, and cesarean delivery for a nonreassuring fetal heart rate pattern was more common in the PPROM associated with meconium-stained amniotic fluid group. Because of the 7-year span of this study, there was not a uniform approach to cervicovaginal cultures or the administration of intrapartum antibiotics. However, after controlling for potential confounding variables, we found that meconium in the amniotic fluid at the time of ruptured membranes remained a marker for early onset neonatal sepsis.

Previous reports have suggested an association between the presence of meconium and infectious neonatal morbidity in the preterm infant. In one report, neonatal sepsis was found to be increased in premature infants who were born to febrile mothers with the presence of meconium in the amniotic fluid.[23] The organism most often associated with sepsis was *Escherichia coli*. In another report linking meconium-stained amniotic fluid to perinatal infection, the infectious outcomes were mostly enteritis, primarily caused by *Streptococcus* (group not specified).[9] Meconium staining of the amniotic fluid has also been reported to be associated with intraamniotic infection in patients with preterm labor who had amniocenteses performed for amniotic fluid cultures.[7] A marked association also has been found between the presence of *L. monocytogenes* and meconium-stained amniotic fluid[8]; however, in our study, *L. monocytogenes* was not isolated from maternal or neonatal cultures. To the best of our knowledge, ours is the first study specifically linking the presence of meconium in amniotic fluid to GBBS neonatal sepsis.

The mechanism through which intrauterine fetal infection may be associated with meconium passage is not well defined. The presence of meconium may predispose to bacterial overgrowth. In vitro studies have found that meconium enhances bacterial growth in amniotic fluid and impairs the natural antibacterial properties of the amniotic fluid.[10,24] In a study focused on GBBS, meconium specifically promoted the growth of GBBS in amniotic fluid.[25] Alternatively, an established infection may precipitate the passage of meconium. The preterm fetus, as opposed to the term fetus, is at risk for acquiring infections from even a relatively low colony count[26] and the presence of meconium may increase this risk. It should be noted, however, based upon mid-trimester amniocentesis data, that the presence of green-stained amniotic fluid may be the result of contamination of the amniotic fluid with hemoglobin and its catabolic products.[27]

Our findings suggest that pregnancies complicated by PPROM associated with meconium-stained amniotic fluid prior to the onset of labor are at increased risk for neonatal infectious morbidity. This association was particularly seen in pregnancies <32 weeks gestation and approached significance in those between 32 and 36 weeks gestation. In addition, these pregnancies are more likely to be complicated by chorioamnionitis, abnormal fetal heart rate patterns, lower Apgar scores and umbilical cord arterial pH, and FGR.

After controlling for potentially confounding variables, we found that meconium remained a marker for early onset neonatal sepsis, with an odds ratio of 11.3. Consequenty, pregnancies complicated by PPROM associated with meconium-stained amniotic fluid should be considered at high risk for neonatal sepsis and may benefit from early administration of antibiotics begun on admission and continued through delivery. In the case of proven pulmonary maturity induction of labor may be indicated to reduce fetal exposure to infection in utero. Prospective studies will be needed to address these issues.

### REFERENCES

1. McGregor JA, French JI, Seo K: Antimicrobial therapy in preterm premature rupture of membranes: Results of a prospective, double-blind, placebo-controlled trial of erythromycin. Am J Obstet Gynecol 165:632–640, 1991.
2. Shubert PJ, Diss E, Iams JD: Etiology of preterm premature rupture of membranes. Obstet Gynecol Clin North Am 19:251–263, 1992.
3. Regan JA, Chao S, James LS: Premature rupture of membranes, preterm delivery, and group B streptococcal colonization of mothers. Am J Obstet Gynecol 141:184–186, 1981.
4. Alger LS, Lovchik JC, Hebel JR, Blackmon LR, Creshaw MC: The association of *Chlamydia trachomatis, Neisseria gonorrhoeae,* and group B streptococci with preterm rupture of membranes and pregnancy outcome. Am J Obstet Gynecol 159:397–404, 1988.
5. Thomsen AC, Morup L, Hansen KB: Antibiotic elimination of group B streptococci in urine in prevention of preterm labour. Lancet 1:591–593, 1987.
6. Katz VL, Bowes WA Jr: Meconium aspiration syndrome: Reflections on a murky subject. Am J Obstet Gynecol 166:171–183, 1992.
7. Romero R, Hanaoka S, Mazor M, et al.: Meconium-stained amniotic fluid: A risk factor for microbial invasion of the amniotic cavity. Am J Obstet Gynecol 164: 859–862, 1991.
8. Halliday HL, Hirata T: Perinatal listeriosis: A review of twelve patients. Am J Obstet Gynecol 133:405–410, 1979.
9. Blot P, Milliez J, Breart G, et al.: Fetal tachycardia and meconium staining: A sign of fetal infection. Int J Gynaecol Obstet 21:189–194, 1983.
10. Florman AL, Teubner D: Enhancement of bacterial growth in amniotic fluid by meconium. J Pediatr 74: 111–114, 1969.
11. Brenner WE, Edelman DA, Hendricks CH: A standard of fetal growth for the United States of America. Am J Obstet Gynecol 126:555–564, 1976.
12. Papile LA, Burstein J, Burstein R, Koffler HO: Incidence and evolution of subependymal intraventricular hemorrhage: A study of infants with birth weights less than 1,500 grams. J Pediatr 92:529–534, 1978.
13. Boyer KM, Gotoff SP: Prevention of early-onset neonatal group B streptococcal disease with selective intrapartum chemoprophylaxis. N Engl J Med 314:1665–1669, 1986.
14. Boyer KM, Gadzala CA, Burd LI, Fisher DE, Paton JB, Gotoff SP: Selective intrapartum chemoprophylaxis of neonatal group B streptoccocal early-onset disease. I. Epidemiologic rationale. J Infect Dis 148:795–801, 1983.
15. Gerdes JS: Clinicopathologic approach to the diagnosis of neonatal sepsis. Clin Perinatol 18:361–381, 1991.
16. Silver HM, Gibbs RS, Gray BM, Dillon HC: Risk factors for perinatal group B streptococcal disease after amniotic fluid colonization. Am J Obstet Gynecol 163: 19–25, 1990.
17. Morales WJ, Angel JL, O'Brien WF, Knuppel RA: Use of ampicillin and corticosteroids in premature rupture of membranes: A randomized study. Obstet Gynecol 73: 721–726, 1989.
18. Mercer BM, Moretti ML, Prevost RR, Sibai BM: Erythromycin therapy in preterm premature rupture of the membranes: A prospective, randomized trial of 220 patients. Am J Obstet Gynecol 166:794–802, 1992.
19. Johnston MM, Sanchez-Ramos L, Vaughn AJ, Todd HW, Benrubi GI: Antibiotic therapy in preterm premature rupture of membranes: A randomized, prospective, double-blind trial. Am J Obstet Gynecol 163:743–747, 1990.
20. Ernest JM, Givner LB: A prospective, randomized, placebo-controlled trial of penicillin in preterm premature rupture of membranes. Am J Obstet Gynecol 170:516–521, 1994.
21. Baker CJ: Summary of the workshop on perinatal infections due to group B streptococcus. J Infect dis 136:137–152, 1977.
22. Schuchat A, Oxtoby M, Cochi S, et al.: Population-based risk factors for neonatal group B streptococcal disease: Results of a cohort study in metropolitan Atlanta. J Infect Dis 162:672–677, 1990.
23. Knudsen FU, Steinrud J: Septicaemia of the newborn, associated with ruptured foetal membranes, discoloured amniotic fluid or maternal fever. Acta Paediatr Scand 65:725–731, 1976.
24. Larsen B, Galask RP: Host resistance to intraamniotic infection. Obstet Gynecol Surv 30:675–691, 1975.
25. Hoskins IA, Hemmings VG, Johnson TRB, Winkel CA: Effects of alterations of zinc-to-phosphorus ratios and meconium content on group B streptococcus growth in human amniotic fluid in vitro. Am J Obstet Gynecol 157:770–773, 1987.
26. Morales WJ, Lim D: Reduction of group B streptococcal maternal and neonatal infections in preterm pregnancies with premature rupture of membranes through a rapid identification test. Am J Obstet Gynecol 157:13–16, 1987.
27. Hankins GDV, Rowe J, Quirk JG, Trubey R, Strickland DM: Significance of brown and/or green amniotic fluid at the time of second trimester genetic amniocentesis. Obstet Gynecol 64:354–358, 1984.

## References

[PDF_00450] Alger L. S., Lovchik J. C., Hebel J. R., Blackmon L. R., Crenshaw M. C. (1988). The association of Chlamydia trachomatis, Neisseria gonorrhoeae, and group B streptococci with preterm rupture of the membranes and pregnancy outcome.. Am J Obstet Gynecol.

[PDF_00468] Blot P., Milliez J., Breart G., Vige P., Nessmann C., Onufryk J. P., Dendrinos S., Sureau C. (1983). Fetal tachycardia and meconium staining: a sign of fetal infection.. Int J Gynaecol Obstet.

[PDF_00486] Boyer K. M., Gadzala C. A., Burd L. I., Fisher D. E., Paton J. B., Gotoff S. P. (1983). Selective intrapartum chemoprophylaxis of neonatal group B streptococcal early-onset disease. I. Epidemiologic rationale.. J Infect Dis.

[PDF_00481] Boyer K. M., Gotoff S. P. (1986). Prevention of early-onset neonatal group B streptococcal disease with selective intrapartum chemoprophylaxis.. N Engl J Med.

[PDF_00474] Brenner W. E., Edelman D. A., Hendricks C. H. (1976). A standard of fetal growth for the United States of America.. Am J Obstet Gynecol.

[PDF_00509] Ernest J. M., Givner L. B. (1994). A prospective, randomized, placebo-controlled trial of penicillin in preterm premature rupture of membranes.. Am J Obstet Gynecol.

[PDF_00471] Florman A. L., Teubner D. (1969). Enhancement of bacterial growth in amniotic fluid by meconium.. J Pediatr.

[PDF_00490] Gerdes J. S. (1991). Clinicopathologic approach to the diagnosis of neonatal sepsis.. Clin Perinatol.

[PDF_00465] Halliday H. L., Hirata T. (1979). Perinatal listeriosis--a review of twelve patients.. Am J Obstet Gynecol.

[PDF_00526] Hoskins I. A., Hemming V. G., Johnson T. R., Winkel C. A. (1987). Effects of alterations of zinc-to-phosphorus ratios and meconium content on group B Streptococcus growth in human amniotic fluid in vitro.. Am J Obstet Gynecol.

[PDF_00504] Johnston M. M., Sanchez-Ramos L., Vaughn A. J., Todd M. W., Benrubi G. I. (1990). Antibiotic therapy in preterm premature rupture of membranes: a randomized, prospective, double-blind trial.. Am J Obstet Gynecol.

[PDF_00458] Katz V. L., Bowes W. A. (1992). Meconium aspiration syndrome: reflections on a murky subject.. Am J Obstet Gynecol.

[PDF_00520] Knudsen F. U., Steinrud J. (1976). Septicaemia of the newborn, associated with ruptured foetal membranes, discoloured amniotic fluid or maternal fever.. Acta Paediatr Scand.

[PDF_00524] Larsen B., Galask R. P. (1975). Host resistance to intraamniotic infection.. Obstet Gynecol Surv.

[PDF_00439] McGregor J. A., French J. I., Seo K. (1991). Antimicrobial therapy in preterm premature rupture of membranes: results of a prospective, double-blind, placebo-controlled trial of erythromycin.. Am J Obstet Gynecol.

[PDF_00500] Mercer B. M., Moretti M. L., Prevost R. R., Sibai B. M. (1992). Erythromycin therapy in preterm premature rupture of the membranes: a prospective, randomized trial of 220 patients.. Am J Obstet Gynecol.

[PDF_00496] Morales W. J., Angel J. L., O'Brien W. F., Knuppel R. A. (1989). Use of ampicillin and corticosteroids in premature rupture of membranes: a randomized study.. Obstet Gynecol.

[PDF_00531] Morales W. J., Lim D. (1987). Reduction of group B streptococcal maternal and neonatal infections in preterm pregnancies with premature rupture of membranes through a rapid identification test.. Am J Obstet Gynecol.

[PDF_00477] Papile L. A., Burstein J., Burstein R., Koffler H. (1978). Incidence and evolution of subependymal and intraventricular hemorrhage: a study of infants with birth weights less than 1,500 gm.. J Pediatr.

[PDF_00446] Regan J. A., Chao S., James L. S. (1981). Premature rupture of membranes, preterm delivery, and group B streptococcal colonization of mothers.. Am J Obstet Gynecol.

[PDF_00461] Romero R., Hanaoka S., Mazor M., Athanassiadis A. P., Callahan R., Hsu Y. C., Avila C., Nores J., Jimenez C. (1991). Meconium-stained amniotic fluid: a risk factor for microbial invasion of the amniotic cavity.. Am J Obstet Gynecol.

[PDF_00516] Schuchat A., Oxtoby M., Cochi S., Sikes R. K., Hightower A., Plikaytis B., Broome C. V. (1990). Population-based risk factors for neonatal group B streptococcal disease: results of a cohort study in metropolitan Atlanta.. J Infect Dis.

[PDF_00443] Shubert P. J., Diss E., Iams J. D. (1992). Etiology of preterm premature rupture of membranes.. Obstet Gynecol Clin North Am.

[PDF_00492] Silver H. M., Gibbs R. S., Gray B. M., Dillon H. C. (1990). Risk factors for perinatal group B streptococcal disease after amniotic fluid colonization.. Am J Obstet Gynecol.

[PDF_00455] Thomsen A. C., Mørup L., Hansen K. B. (1987). Antibiotic elimination of group-B streptococci in urine in prevention of preterm labour.. Lancet.

